# Evaluation of the anticancer potential of a sulphonamide carbonic anhydrase IX inhibitor on cervical cancer cells

**DOI:** 10.1080/14756366.2019.1579805

**Published:** 2019-02-27

**Authors:** Ismail Koyuncu, Yasin Tülüce, Hewa Slahaddin Qadir, Mustafa Durgun, Claudiu T. Supuran

**Affiliations:** a Department of Biochemistry, Faculty of Medicine, Harran University, Sanliurfa, Turkey;; b Department of Medical Biology, Faculty of Medicine, Van Yuzuncu Yil University, Van, Turkey;; c Department of Chemistry, Faculty of Arts and Sciences, Harran University, Sanliurfa, Turkey;; d Neurofarba Department, Section of Pharmaceutical and Nutriceutical Sciences, Università degli Studi di Firenze, Sesto Fiorentino (Florence), Italy

**Keywords:** Apoptosis, cytotoxicity, cervical cancer, carbonic anhydrase-IX inhibitor, oxidative stress

## Abstract

Cervical cancer is a common type of cancer. Carbonic anhydrase IX (CA IX) is an attractive target for tumour therapy, being overexpressed in many cancers. We investigated the anticancer properties of the aromatic sulphonamide S-1 as a CA IX inhibitor on cervical cancer cells (HeLa) positive for CA IX expression and normal prostate epithelial cell line (PNT1-A) negative for CA IX. We examined the cytotoxic, apoptosis, genotoxic, and oxidative stress activity of S-1 on HeLa and PNT1-A cell lines. S-1 induced significant reduction of cell viability, caused apoptosis, and up-regulated ROS production. This decrease in cell survival rate can be attributed to the high level of ROS and apoptosis, which has also been shown to arrest the cell cycle. Our findings indicated that S-1 is more effective on HeLa than PNT1-A. S-1 was able to induce apoptosis of cervical cancer cells and is a possible candidate for future anticancer studies.

## 1. Introduction

Cancer is a disease characterised by the continuous growth of whole cells due to different causes and factors, it was considered as one of the reasons that led to deaths in the modern life, it has been estimated as the second leading infection that causes death worldwide after cardiovascular diseases^1^. Most of the global cancer cases have been reported with 63% mortality in developing countries^2^. Cervical cancer (CC) is one of the widespread cancer that is present in females, living in low- and middle-income countries. It is the third most prevalent gynecologic cancer diagnosed and source of death in the United States^3^. The main factor responsible for CC is the viral infections, HPV E6 and E7 are inhibitory proteins expressed by the human papillomavirus (HPV) that liable for the genesis of the cervical neoplasm that HeLa cells are obtained^4^. As a result of inactivation of P53 by HPV E6, the cell cycle regulation is disrupted; HPV E7 promotes Rb protein degradation and leads to uncontrolled cell division^5^. These two inhibitory proteins are responsible for the proliferation of HeLa cells indefinitely by inhibiting the formation of apoptosis^6^. In a given biological compartment, apoptosis was undertaken arrangement task to sustaining tissue homeostasis by mediating the balance between cellular depletion and cellular increment. Morphological features of apoptosis include membrane budding, cell crumpling, chromatin condensation, and nucleosomal degradation. The intrinsic and extrinsic pathways regulating Apoptosis, which is considered to be the mechanism of cell death caused by chemotherapy, are the focus of many preclinical drug discovery researches^7–9^. The internal pathway is generally activated as a consequence of cellular response to intracellular stress signals such as DNA damage, rising reactive oxygen species (ROS), viral infection, and activation of oncogenes. The other pathway, the extrinsic one, is stimulated as a consequence of the integration of the extracellular ligand into the corresponding receptor located on the plasma membrane.

Both of the above-mentioned pathways activate the proteolytic enzymes called Cysteinyl aspartate-specific protease (Caspase), which in turn mediate the rapid disassembly of cellular structures and organelles. Caspases are a family of proteins that have a nucleophilic cysteine residue that participates in the cleavage of aspartic amino acid-containing moieties of protein biomolecules^10^. Cytotoxicity is the result of molecular events leading to the inhibition of the expression of a variety of macromolecules, and consequence of significant damage to the function and structure of the cell^11^. Chemotherapeutic drugs are targeting apoptosis pathways to induce cancer cells to cell death programme. The cytotoxic effect of chemotherapy on cancer cells can result in DNA damage, blocking genetic materials, and increase ROS in these cells that are affected by chemo drugs, the chemotherapy drug that induces to increase ROS in cancer cells play as decreased anti-oxidant. ROS induce cytochrome c (cyt c) release from mitochondria to the cytoplasm, to activate caspase 3 and resulted from a membrane permeability transition and cancer cells via an apoptosis pathway and killed as Emodin drug. As a chemotherapeutic drug that targets apoptosis, cisplatin leads to apoptosis in cancer cells through DNA damage, which is caused by cross-linking with purine bases. Medications are used to treat cervical cancer, despite serious side effects such as kidney problems, allergies, hemorrhage^12^. The carbonic anhydrase (CA) family contains 16 metalloenzymes, which catalyse the hydration of CO_2_ to bicarbonate and H^+^ with catalytically active zinc^13^. These isoenzymes differ mainly in terms of catalytic activity, tissue distribution and intracellular localisation. Indeed, cytosolic, mitochondrial, secreted, and membrane-bound isozymes have been identified^14^. CAs are found in a variety of tissues such as the gastrointestinal tract, the reproductive system, the nervous system, the kidneys, the lungs, the skin and the eyes^15^ ^,^ ^16^. Many CA isoforms are involved in critical physiologic processes such as respiration and acid-base regulation, electrolyte secretion, bone resorption, calcification and biosynthetic reactions which require bicarbonate as a substrate. Two CA isozymes (CA IX) that are evidently over-expressed in most tumours are involved in critical processes linked to cancer progression and response to treatment^15^. CA IX is naturally expressed in only a few normal tissues (stomach and body cavity lining), but it is ectopically induced and highly overexpressed in many solid tumour types^15^ ^,^ ^17^. Therefore, the researchers looking for, discovering new drugs that more effective on cancer, are not stopping till now. Researchers are constantly working towards developing renovated medicines that selectively kill tumour cells and protect healthy tissue with less many side effects. Additionally, molecules should have a protective effect against toxic substances in healthy cells^18^.

In a previous study, we have reported the synthesis and inhibitory activity of S-1 compound against carbonic anhydrase isoforms I, II, IX and XII ^19^. Furthermore, the cytotoxic effects of similar sulphonamide compounds were examined on several cancer cell lines as well as normal cells^20^. In this study, two cell lines and two chemicals have been used, CC cell line (HeLa) as the target cell and PNT1-A as control, while the chemicals used as anticancer agents were S-1 as a new agent and cisplatin as a control. The aim of this study is to estimate the level of cell viability, programmed cell death, and DNA damage effects of S-1 by determining the cytotoxicity, apoptosis, and cell cycle assay on HeLa cell line. In addition, total oxidant status (TOS), total antioxidant status (TAS) and oxidative status index (OSI) were measured.

## 2. Materials and methods

### 2.1. Cell culture

HeLa and PNT1-A were cultured in RPMI-1640 medium supplemented with 10% Foetal bovine serum (FBS) and penicillin (100 U/mL)/streptomycin (100 mg/mL), L-glutamine (2 mM) at 37 °C in a 5% CO_2_ humidified atmosphere. Since it is an unstable amino acid, the medium was supplemented with L-glutamine immediately prior.

When cells were grown to a sufficient number, they were separated with sterilised trypsin/EDTA solution. As the cells were detached in 37 °C incubator, more complete medium was added to inhibit the residual trypsin activity. Cells were counted with a haemocytometer by the following formula:

Cells/ml=average count per square×dilution factor×10

Equal volumes of cell suspension were seeded onto new dishes at appropriate densities.

### 2.2. Novel aromatic sulfonamide derivative (S-1) as a CAIX inhibitor

**Figure 1.**
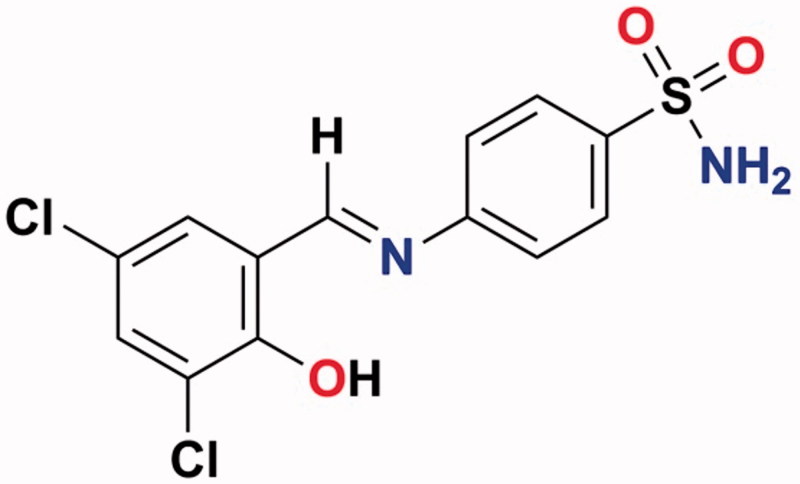
The chemical structure of sulphonamide S-1.

The design of isoform-selective CA inhibitors could lead to uncovering drugs with fewer side effects compared to the currently used ones^21^. In this study, a sulphonamide derivative S-1 was used as a CAIX inhibitor. The sulphonamide derivative, 4-[(3,5-Dichloro-2-hydroxybenzylidene)amino]benzenesulfonamide (S-1) was obtained by condensation of sulphanilamide with 3,5-dichloro-2-hydroxybenzaldehyde and characterised using both analytical and spectroscopic methods. Some data for compound S-1: Yield: 85%; Colour: Bright red; M.p.: 242–244 °C; Anal. Calcd. for C_13_H_10_Cl_2_N_2_O_3_S (345.20 g/mol), LC–MS/MS (*m*/*z*): 342.95 [M − H] (Figure 1)^19^. The sulfonamide compound (S-1) containing the imine bond is stable in the assay medium.

### 2.3. Cell viability assay (WST-1) and IC_50_ measurement

WST-1 Cell Proliferation test, a product of the Roche Company is a colourimetric assay based on the cleavage of a tetrazolium salt by mitochondrial dehydrogenases to generate formazan in living cells. This assay is simple because only 10 μL of WST-1 reactant is added to 100 μL media on a 96-well plate. The cells were counted with a haemocytometer immediately before the subsequent procedures. The HeLa and PNT1A cells were used for seeding, with 5 × 10^3^ cells per well, and then incubated overnight in a humidified atmosphere (37 °C with 5% CO_2_).

The next day, WST-1 is added to cells treated with S-1 as a cytotoxic reagent and the incubation is continued for an additional 1−2 h in a humidified atmosphere. One of the advantages of the WST-1 test is that colour change (yellow) can be seen with the naked eye.

After incubation, the plate was shaken well for 1 min. The absorbance is then measured at 450 nm versus a 650 nm reference by using a plate reader (M5, Spectra MAX) and SoftMax Pro software from Molecular Devices to compare experimental samples to controls.

IC_50_ is a measure used to estimate the potency of a material that inhibits a specific biological or biochemical function. According to the anti-proliferative effects of S-1, the half maximal inhibitory concentration IC_50_ value for HeLa cells treated with S-1 was perceived as much lower compared to PNT1-A cells.

### 2.4. Apoptosis assay

This protocol was developed to directly determine the percentage of early and late apoptotic cell populations induced in cultures. The appropriate numbers of cells required for the apoptosis test were produced and thus the number of viable, dead, early, and late apoptotic cells were determined.

Annexin V is a calcium-dependent phospholipid binding protein having a high affinity for phosphatidylserine (PS), a membrane component that is normally localised to the inner surface of the cell membrane^22^. At the beginning of the apoptotic pathway, PS molecules translocate to the outer surface of the cell membrane to which Annexin V can be easily attached^23^.

Culture cells containing HeLa and PNT1-A controls for a suitable time to induce apoptosis were prepared for incubation with Annexin V reagent. The Annexin V reagent was allowed to warm up to room temperature, each tube was supplemented with a 100 μL cells. In performing this test, cells must be in suspension with at least 1% blocking solution BSA, 1%, FBS, or 10% filtered NHS. Then 100 μL of Annexin V reactant was added to each of these tubes. The solution was thoroughly vortexed at medium speed for 3 to 5 s and the stain samples were left in the dark for 20 min at room temperature. Annexin V assays were performed on a Cell Analyser allowing quantitative analysis of live, early and late apoptosis and cell death in both adherent and suspension cell lines.

### 2.5. Cell cycle assay

Cell cycle analysis has become increasingly important in understanding the effect of anti-cancer compounds^24^ or in examining cell division mechanisms. The Cell Cycle Kit provides an easy and fast quantitative estimate of the cell percentage in the G0/G1, S and G2/M phases of the cell cycle by the Cell Analyser.

Cell cycle test uses a premixed reagent containing RNase A, as well as propidium iodide (PI), which binds to the DNA by entering the bases in a particular formulation. A kit produced by the manufacturer of the Cell Analyser (Cat No MCH 100106) was used to determine the potential for arresting the cell cycle of the novel aromatic sulphonamide derivative. The cells were collected and counted after the S-1 treatment was completed. Approximately 12 × 10^4^ cells from control and treated groups were fixed in 75% ethyl alcohol for 3 h at −20 °C. Furthermore, cells were washed once with PBS and incubated with 200 μL of assay solution for 30 min at room temperature. After completion of the incubation period, the cells were vortexed gently and assayed in a cell analyser. Cell numbers at each point in the cell cycle such as G0/G1, S and G2/M phases of S-1 treated cells, including control, were analysed.

### 2.6. Intracellular TAS determination

The cellular TAS amount was determined using a new colourimetric assay method, which was improved by Erel^25^. This process is withstanding on the bleaching of the ostensible colour of the 2,2′-azino-bis [3-ethylbenzothiazoline-6-sulfonic acid] (ABTS) radical cation via the action of antioxidants. Upon the addition of a sample, the oxidative reactions initiated by the hydroxyl radicals present in the reaction mixture are inhibited by the antioxidant components of the lysate, preventing colour change and thus providing an effective measure of the TAS value of the lysate. The precision of this test has high accuracy (less than 3% error rate). Analysis results were expressed as mmol Trolox equiv/mg protein.

### 2.7. Intracellular TOS determination

The amount of cellular TOS was measured using an automated colourimetric reading process advanced by Erel^26^. In this method, the oxidation reaction is increased by the presence of excess glycerol in the reaction medium. Ferric ion forms a coloured complex with chromogen orange in an acidic environment. The spectrophotometrically measurable colour intensity is concerned to the whole level of oxidant molecules existent in the specimen. The assay is calibrated with H_2_O_2_, and the results are expressed in terms of micromolar H_2_O_2_ per mg protein (μmol H_2_O_2_ Equiv./mg protein).

### 2.8. Oxidative stress index

Percentage of TOS to TAS amount is taken into account in order to determine the oxidative stress index (OSI)^27^. Oxidative stress index was determined according to the equation outlined below:

OSI = (TOS (μ mol H_2_O_2_ equiv/g))/(TAS (μ mol Trolox equiv/g)) × 10

### 2.9. Statistical analysis

All outcomes, including the measured concentrations of apoptosis, WST-1, Cell phases rate, TAS, TOS, OSI were evaluated statistically and the final results were expressed as mean ± SD. The results were achieved by taking the average of three replicates. All data from the experiments were analysed using variance analysis (one-way ANOVA) of the MINITAB packet programme adapted for Windows. The *p* < .05 was accepted as statistically significant.

## 3. Results

### 3.1. Effect of S-1 on cell proliferation

**Figure 2.**
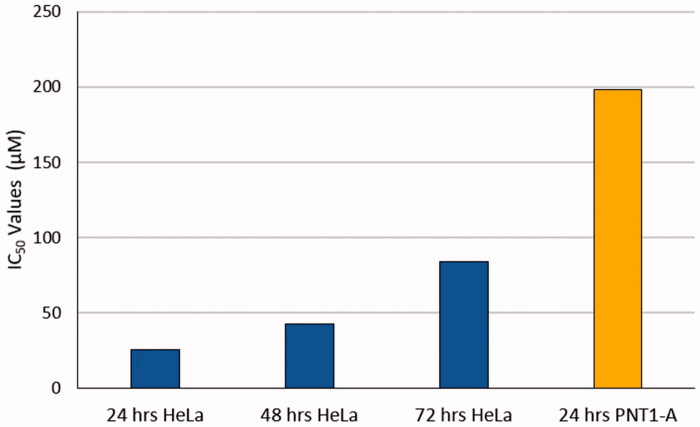
Provides the IC_50_ value of S-1 on HeLa and PNT1-A cell lines (*p* < .05).

The antiproliferative effect of a synthesised sulphonamide derivative S-1 has been observed in two different cell lines, CC cell line HeLa, and human adult normal prostatic epithelial cell PNT1-A. The IC_50_ value, considered as the half maximal inhibitory concentration in HeLa cells applied S-1, is much smaller than that of the other PNT1-A cells administered S-1. As can be seen in Table 1, IC_50_ is a measure used to estimate the capacity of the S-1 that inhibits the cancer cell's survival effort. The smaller the Ki value represents the minimum amount of S-1 required to inhibit CAIX enzyme activity, while demonstrates a large binding affinity. In an *in vitro* study, IC_50_ symbolises the concentration of S-1 that is required for 50% inhibition. Figure 2 clearly shows that the sensitivity of the HeLa cell line to S-1 is greater than that of PNT1-A cell line.

**Table 1.** IC_50_ values of S-1 on HeLa and PNT1-A cell lines and Ki of CAIX (*p* < .05).

Cell line	IC50 values		
	S-1	AAZ	Cisplatin
HeLa 24 h	25.2 µM	52.3 µM	28.9 µM
HeLa 48 h	42.9 µM	45.3 µM	7.6 µM
HeLa 72 h	84.1 µM	54.3 µM	6.1 µM
PNT1-A 24 h	198.2 µM	72.3 µM	13.4 µM
Ki value of CA-IX	35.6 nM	25.0 nM	**–**
Ki value of CA-XII	7.5 nM	6 nM	**–**

In this study, CA IX inhibitory effects are generally studied in HeLa cell since the CA-IX expression level in this cells is higher than that of hCA XII and CA XII. Therefore, the mechanism of action of CA XII inhibition effect on HeLa cytotoxicity was not studied. In addition, the effect of S-1 on normal cells is lower than 5-FU and AAZ (Acetazolamide) which are used as controls, and it increases the possibility of cytotoxic effect only due to CA-IX inhibition (Table 1). Previous studies have shown that CA-IX inhibitors, sulfonamide compounds, act on HeLa cells with high CA-IX expression levels^20^.

**Figure 3.**
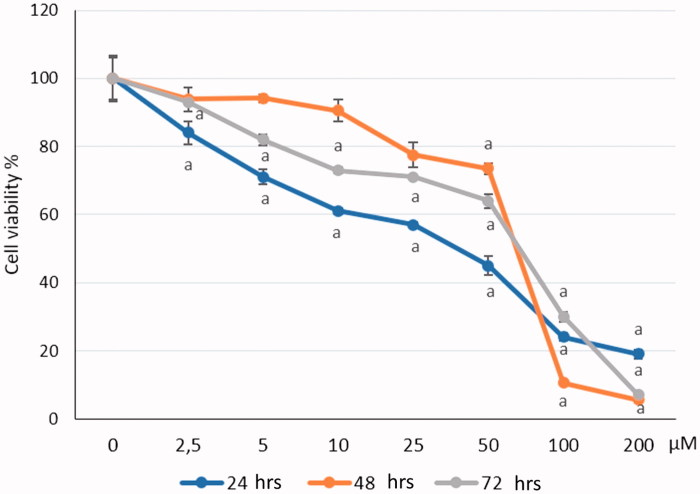
Cytotoxic effect of different doses of S-1 on HeLa cell line. (a) *p* values <.5 compared with negative control.

**Figure 4.**
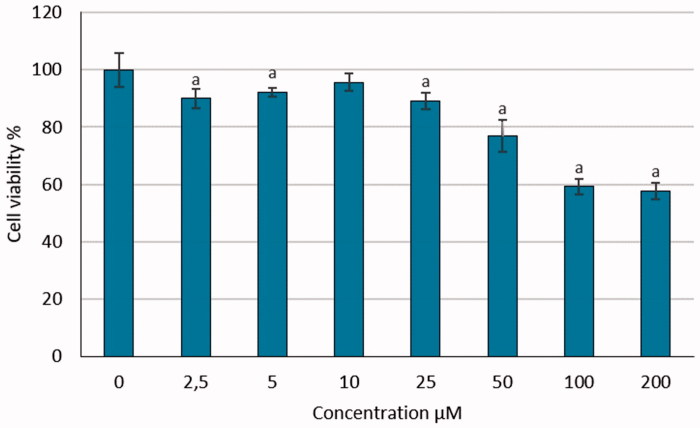
Cytotoxic effect of different doses of S-1 on PNT1-A cell line. (a): *p* values <.05 compared with 0 dose.

We determined the cell viability of CC cell line HeLa grown in 96-well plates for 24, 48, 72 h and treated with increasing concentrations (0, 2.5, 5, 10, 25, 50, 100, 200 μM) of S-1. Cell viability was evaluated with colourimetric WST-1 assay as described previously tested on the splitting of the tetrazolium salt by mitochondria dehydrogenases in viable cell. The HeLa cells were cultured with different doses of S-1 for 0 to 72 h and then harvested thus obtained findings show the number of viable cells. The number of viable cells was then quantified using trypan blue exclusion. The result of WST-1 showed the effect of S-1 on HeLa cells for the first dose and the increasing effects of S-1 by rising concentration. The greatest viability loss (5.50%) was found at 200 μM for 48 h incubated. The live cells were reduced by the effect of S-1. All data were significant when compared with zero dose (0 μM) as a negative control in 72 h, in time manner *p* values <.05. Figure 3 indicates the cytotoxicity of various concentrations of S-1 in HeLa cells. S-1 exerts cytotoxic and anti-proliferative efficacy on CC cells but own less impact on human healthy PNT1-A cells (Figure 4). This approach with potentially low cytotoxic effects on normal cells may offer a new therapeutic benefit in the treatment of cervical cancer.

### 3.2. Apoptosis detection by Annexin V affinity assay

**Figure 5.**
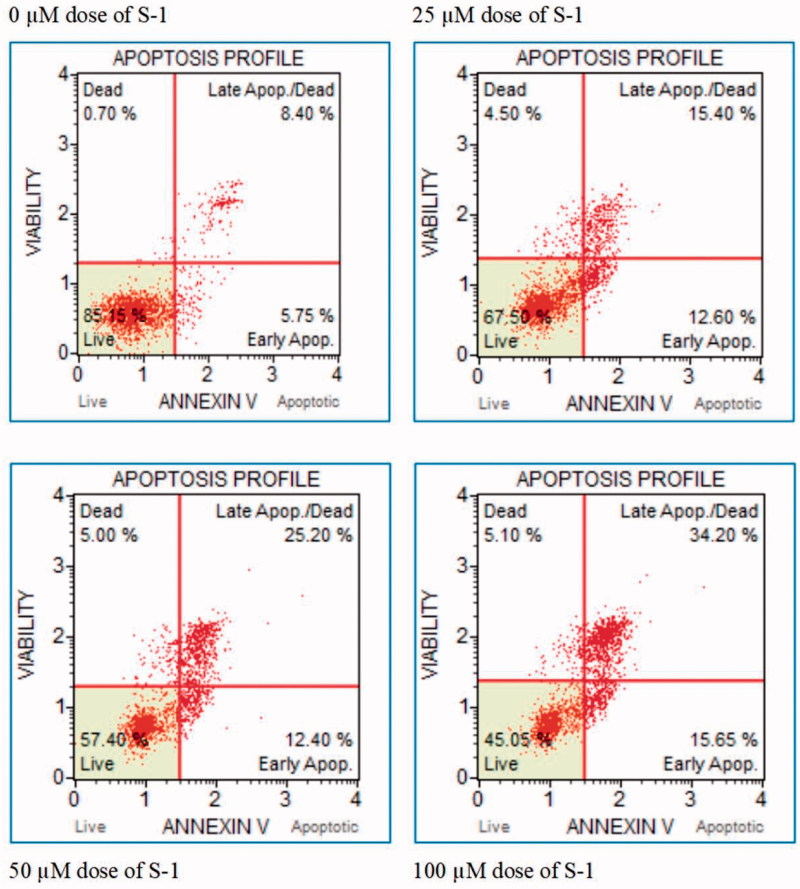
Apoptotic rates of HeLa cells after Annexin V staining.

In order to determine whether various concentrations (20, 50, 100 μM) of S-1 has an effect on apoptosis of HeLa cell line. The Annexin-V test was implemented to gauge apoptosis. Cells were stained by using Annexin-V stain (Figure 5). The method is an effective way to detect apoptosis rate tested on localisation of PS to the outer membrane. In the normal cell, the PS is located in the inner cell membrane, but during apoptosis, the PS is displaced out of the cell membranes.

**Figure 6.**
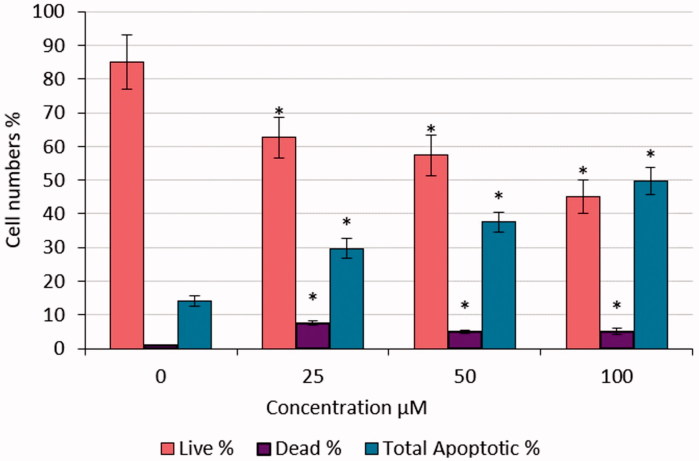
Live, dead and apoptotic rates of HeLa cells treated with S-1. *p < .05.

**Figure 7.**
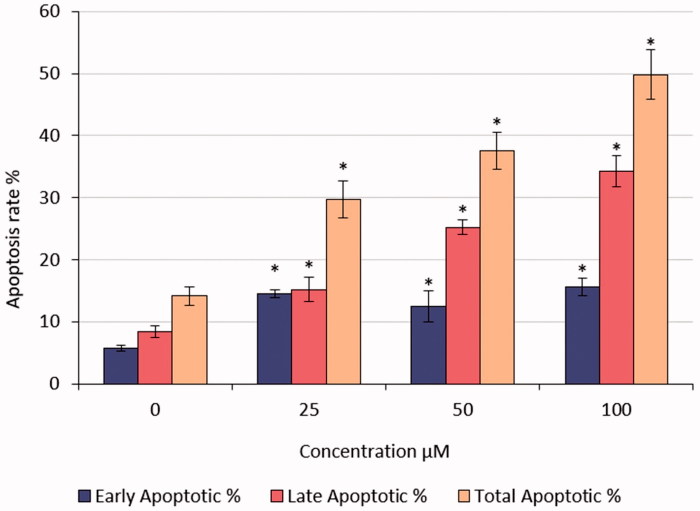
Early, late, and total apoptotic rates of S1-treated HeLa cells. *p < .05.

The percentage of early apoptotic cells was significantly increased compared with negative control (0 μM) (Figures 6 and 7). The number of late apoptotic cells was increased in HeLa cells treated with different doses of S-1 compared with untreated (0 μM) as a negative control. These increases were significant (Figure 6).

Also, after treatment of HeLa cells with S-1, the findings have shown that living cells were decreased and dead cells were elevated by apoptotic pathways. The results were replicated three times and the average was calculated (Figure 6).

### 3.3. Cell cycle test

**Figure 8.**
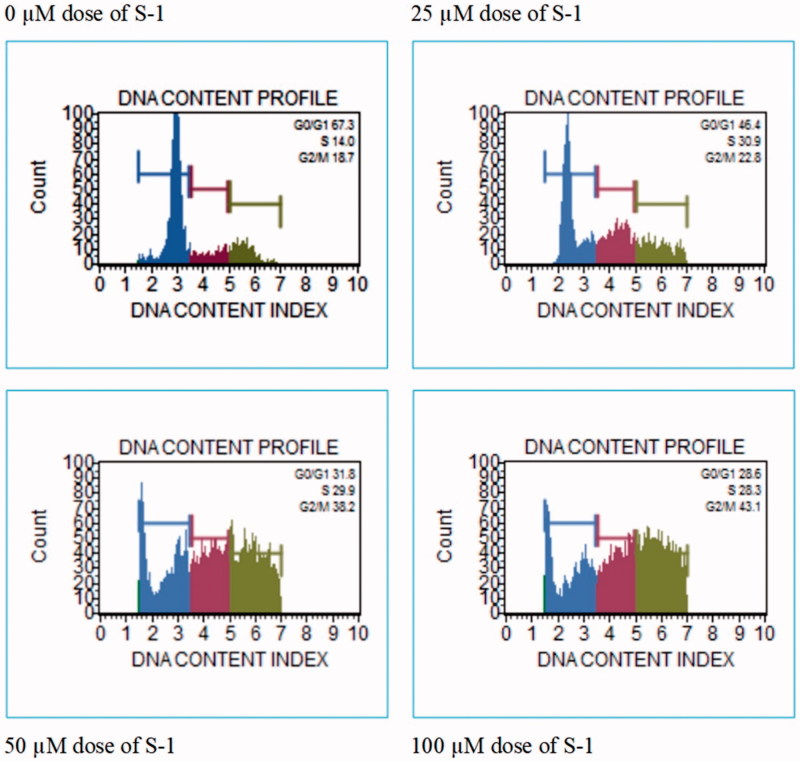
Cell phases rate of HeLa cells treated with S-1.

**Figure 9.**
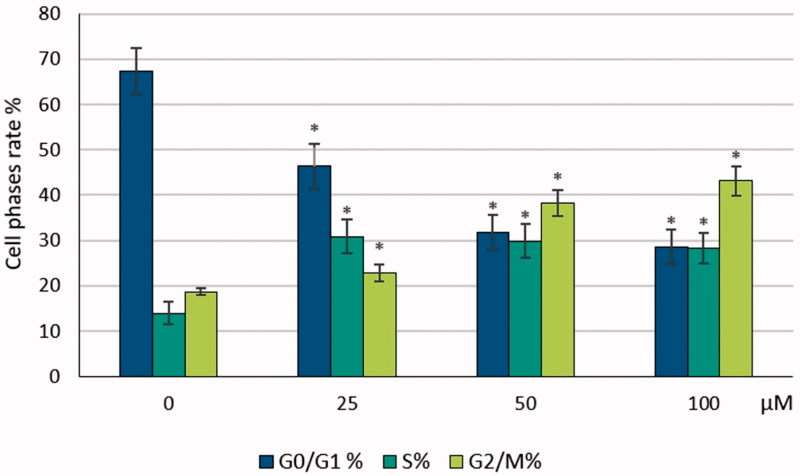
Cell cycle phases of HeLa cells treated with S-1. *p < .05.

We used the cell cycle test kit to determine the effects of S-1 on a related cell cycle of HeLa cell line. The test is shown the cell phases rates, G0/G1, S and G2/M. These phases of cell show the growth and inhibition effects of S-1 on HeLa cells (Figure 8). Our data demonstrated G0/G1 decreased with treatment of by all doses of S-1 respectively. S phase was increased at all doses of S-1. G2/M phases were elevated, rising in all concentrations of S-1 gradually (Figure 9). All data were compared with a dose of 0 µM, which was used as a negative control group in this lab work.

### 3.4. Measurement of TAS, TOS, and OSI values

**Figure 10.**
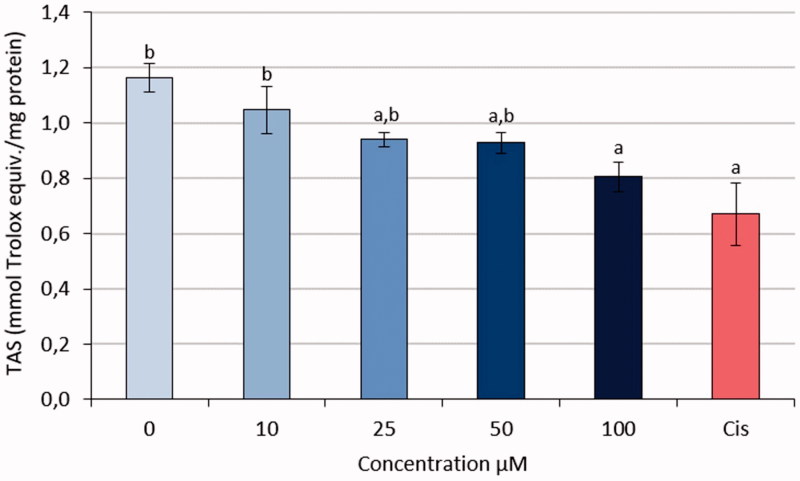
TAS values of HeLa cell line treated with S-1 and CIS. (a): *p*-value <.05 compared with 0 dose, (b) *p*-value <.05 compared with CIS.

The obtained data of HeLa cells administered S-1 at different concentrations showed that the antioxidant ratios decreased. All outcomes were statistically different when compared with 0 doses as negative group. The effect of S-1 in dose 100 µM was similar to Cisplatin (0.67) as a positive control group (Figure 10).

**Figure 11.**
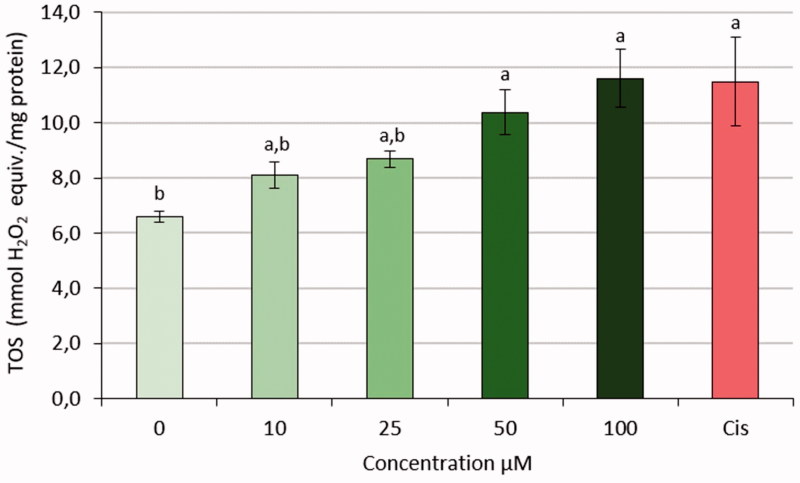
TOS values of HeLa cell line treated with S-1 and CIS. (a): *p-*value <.05 compared with 0 dose, (b) *p*-value <.05 compared with CIS.

The mean of TOS levels of HeLa cells treated with S-1 at different doses was significantly increased, when compared to negative control *p* < .05. Data showed S-1 in doses 50, 100 µM had nearly same effect as Cisplatin (11.49) when compared with positive control groups (Figure 11).

**Figure 12.**
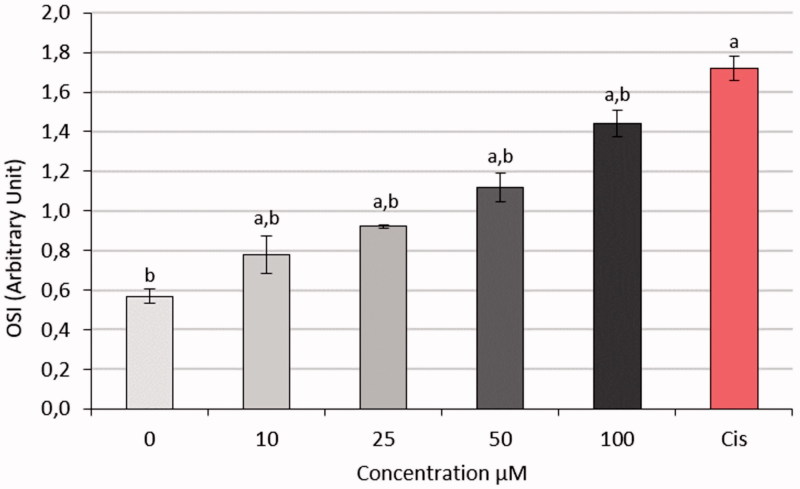
OSI values of HeLa treated with S-1 and CIS. (a): *p*-value <.05 compared with 0 dose, (b) *p* value <.05 compared with CIS.

OSI values were measured based on TOS and TAS values as shown in Figure 12. The OSI levels of the HeLa cell line increased in a dose-dependent manner after increasing doses of S-1. The effect of S-1 at 100 μM concentration applied at the last dose was too close to the level of Cisplatin (1.72).

## 4. Discussion

In this study, the cytotoxic effect of low-dose cytotoxicity in HeLa cells which are CA-IX expression, and the low cytotoxic effect of low CA-IX expression in PNT-1A cells is the most important proof that this substance has a selective effect. It was also supported by molecular techniques such as Annexin V, cell cycle, where the compound exhibited anticancer activity on HeLa cells. Another important finding of the compound-related anticancer activity is the investigation of the effects of oxidative stress which is the secondary effect due to CA-IX inhibition of the mechanism underlying anticancer activity.

Cervical cancer is the name of the illness in which the cells of the cervix become abnormal and multiply so that it cannot be controlled. Cancer chemoprevention refers to the use of substances of natural origin, biological agents, synthetic or chemical compounds to reduce or delay the occurrence carcinogenic progression of tumor^28^.

A comprehensive study of the inhibition mechanisms of carbonic anhydrase inhibitors has opened the way for imaging and treatment associated with carbonic anhydrase^29^. Sulphonamide-based compounds (sulfonamides, sulphanilamides, sulfamates, and their derivatives) are small molecule inhibitors of CAIX isoenzyme that inhibit carbonic anhydrase by coordinating the zinc ion in the active site with the inhibition of μM to nM Ki^30^. Due to its high affinity, availability and ease of chemical manipulation, sulphonamide derivatives can be evaluated as the most potent class of CAIX inhibitors^31^.

Previously, we demonstrated the novel synthesised S-1 attenuated apoptotic, cytotoxic, cell cycle pathways and oxidative stress, therefore, S-1 may have an anti-cancer potential in cervical carcinoma. The antitumor activity of S-1 in HeLa cells and the main components of the mechanism underlying this impact were investigated. An important implication of these findings is the number of viable cells remaining in harvested HeLa cells after culturing with different S-1 doses for 0 to 72 h. S-1 has anti-proliferative efficacy on cervical cancer cells but has less effect on human normal PNT1-A cells. For the first time in the literature, this work has revealed that S-1 exposure reduces cell viability in HeLa cells, induces cell cycle retention and increases cell apoptosis.

According to our result, after treatment of S-1 on Hela cells, it is shown that living cells were decreased and dead cells were elevated by apoptotic pathways. The findings of our research are quite convincing, and thus the following conclusions can be drawn: the synthesised sulfonamide derivative S-1 as a CAIX inhibitor may provide a therapeutic option in cervical carcinoma by selectively targeting apoptotic pathways in the cancerous cervical cell line.

A promising strategy to develop effective chemopreventive and chemotherapeutic approaches in target organ carcinogenesis is to use biochemical differences between cancer cells and normal healthy cells. In this context, selective apoptosis-inducing agents of cancer cells are of great interest as novel cancer prevention options^32^.

Our data demonstrated that G0/G1 decreased when treated with doses of S-1. S phase rose in all the doses of S-1 and showed further increase including rise in G2/M phases. S-1 arrested HeLa cells in G0/G1 phase. These findings are surprising given the fact that another research showed cell cycle stops during oxidative stress^33^. It has been observed that the securinine substance is known to raise the level of ROS in HeLa cells which leads to an increase in the number of autophagic SW480 cells and to retain the cells in the G1 phase of the cell cycle. This causes detriment to biomolecules and can initiate programmed cell death processes through delays in cell cycle control points and deterioration of integrity of mitochondria wrapped with double membrane layer^34^. The cell cycle was also retained in MCF-7 cells, in HCl-60 cells treated with securinine, and in SW480 cells that were incubated with virosecurinine^35^.

This nicely fits into the observation of our data showing that OSI production was significantly increased in cervical cancer. The TAS ratios of S-1 treated HeLa cells dropped down. Oxidative stress, defined as an imbalance between oxidant and antioxidant compounds in the living organism, has been associated with many factors affecting cell survival.

Also, TOS levels of HeLa cells that are treated with different doses of S-1 increased significantly. As a positive control, CIS is a commonly used anticancer drug that has been commonly used in cancer therapy, has the same effect of S-1 as a novel synthesised sulphonamide derivative. Thus, the effects of the oxidative stress of CIS, which consequently reduce the HeLa cell number, support the result of S-1. The apoptosis observed in the present study is due to S-1 administration and it is possible to say that the increase in ROS level is accompanied by the related case. This study is similar to the work done by Ray et al.^34^, which shows that the ROS may be produced during oxidative phosphorylation from mitochondria or may occur during interaction with external sources.

In the current study, apoptosis was significantly rising in early and late apoptosis and dead cells while decreased in living cells. Apoptosis concerns oxidative stress, a condition where there is an imbalance between ROS production and detoxification^36^. ROS is one of the factors that activate the intrinsic pathway of apoptosis and is associated with reduced mitochondrial membrane potential.^37^ When severe oxidative stress occurs, the cell cycle is arrested firstly transient and then permanently, and ROS causes an alteration of membrane permeability, DNA damage^33^ ^,^ ^38–40^.

In conclusion, this study has shown S-1 cytotoxic, genotoxic, and apoptotic activity by increasing ROS production on cells, we compared the cytotoxic activity of synthesised S-1 on HeLa cells. The anti-cancerous potential of the compound not only demonstrated the antiproliferative effect but also induced the apoptotic pathway while maintaining cell cycle arrest. S-1 appears to be the compound with significant anticancer potential. The important point should be emphasised again that S-1 as a novel CAIX inhibitor anticancer effect was investigated for the first time in this study.
